# Addressing Psychosocial Vulnerabilities Through Antenatal Care—Depression, Suicidal Ideation, and Behavior: A Study Among Urban Sri Lankan Women

**DOI:** 10.3389/fpsyt.2021.554808

**Published:** 2021-05-24

**Authors:** Alexis Palfreyman

**Affiliations:** Institute for Global Health, University College London, London, United Kingdom

**Keywords:** antenatal care, pregnancy, depression, Edinburgh Postnatal Depression Scale, suicide, Columbia-Suicide Severity Rating Scale, Sri Lanka

## Abstract

An absence of data persists for common perinatal mental disorders and suicidal ideation and/or behaviors (SIB), particularly from low- and middle-income countries and from the antenatal period. Capitalizing on Sri Lanka's strong antenatal platform, we identify the prevalence of antenatal depressive symptomology, lifetime- and current-pregnancy SIB and their risk factors in women in urbanizing Sri Lanka, and present opportunities for improved antenatal detection of psychosocial vulnerabilities. One thousand antenatal women in Gampaha District from all trimesters of pregnancy were screened in 2016 using a novel three-part instrument, including the validated Edinburgh Postnatal Depression Scale, a modified Columbia-Suicide Severity Rating Scale for first ever use among a perinatal and South Asian population, and an original Life Circumstances questionnaire (with validated subscales). Prevalence and risk factors associated with depressive symptomology and SIB were explored using univariate, bivariate and logistic regression analyses. Women ranged from 16 to 42 years; 46% were nulliparous. Past-week prevalence of antenatal depressive symptomology was high (29.6%). One in four women reported a lifetime history of SIB, while SIB during the current pregnancy was reported at 7.4%. Exposure to intimate partner violence and lifetime SIB emerged as the strongest correlates of both depressive and current-pregnancy SIB outcomes (*p* < 0.05). This study evidences the high prevalence of multiple psychosocial vulnerabilities in pregnant women in Sri Lanka and underscores the need for their improved comprehensive assessment. Given antenatal care's high rates of use in Sri Lanka and in low- and middle-income countries in general, this study presents it as a promising mechanism through which to effectively screen for multiple psychosocial vulnerabilities, supporting early identification and intervention for at-risk women and their families.

## Introduction

Globally, antenatal care (ANC) provides a unique opportunity to identify and support women at risk of poor maternal health outcomes and has demonstrated effectiveness in reducing multiple health and social vulnerabilities. In low- and middle-income countries (LMICs), where most maternal mortality and morbidity occurs (1), ANC may be the first and/or primary mechanism for women to connect with formal health services (2). Maternal health programs continue to focus on obstetric causes of mortality and morbidity. However, more recent evidence suggests common perinatal mental disorders (CPMDs) such as depression and anxiety are the commonest morbidities experienced by perinatal women (3). As a result, programs have missed large subsets of women experiencing CPMDs and—although rarer—symptoms of psychosis, self-harm, and suicide (4). Stakeholders are increasingly looking to introduce or improve mental health screening, referral, and treatment in maternal health services including by non-mental health specialists (5). Several high-income countries have invested in routine screening and/or issued guidance recommending universal screening of perinatal women in primary care (5), albeit with limited screening instruments.

In LMICs, however, perinatal mental health problems remain under-identified and undertreated in part because data on the prevalence and correlates of CPMDs and suicidal ideation and/or behaviors (SIB) are lacking (1, 6), particularly for the antenatal period. By 2016, 20 low- and middle-income countries had published evidence on antenatal depression with half the studies originating from just three countries (Brazil, South Africa and Turkey) (6). Prevalence estimates for CPMDs—which include mood disorders, anxiety, alcohol, and substance abuse—vary widely across contexts and differ depending on when and with what instruments women are screened, how measures are administered (e.g., self-report vs. clinical assessment), and in what environment (e.g., community vs. hospital). In high-income countries, 10 and 13% of ante- and postnatal women experience depression and/or anxiety, respectively (1). Limited LMIC evidence suggests perinatal women experience double the prevalence of CPMDs compared to their high-income country counterparts, with 16–25% antenatal and 20% postnatal prevalence, respectively (1). Across all settings, antenatal depression is a recognized predictor of postnatal depression (1, 6).

As obstetric causes of maternal death have fallen, deaths due to suicide have emerged as significant if not leading contributors to preventable deaths in perinatal women, including during pregnancy (3, 6). With the postnatal period too late for intervention, there is an urgent need to identify antenatal women at risk of SIB. Existing data are rare, however, and similarly weighted by evidence from high-income contexts (6, 7). Maternal suicides reflect a double disparity in LMIC where local evidence is most limited, but incidence is highest. In LMICs, where 79% of all suicides occur (8), a pooled prevalence rate of between 0.65 and 3.55% of maternal deaths is attributed to suicide (9). Further, suicide represents only part of SIB, which also encompasses suicidal thoughts, planning and preparatory behaviors, and suicide attempts. Most research on perinatal women has focused on suicidal ideation, with global prevalence estimates ranging from 5.0 to 27.5% (4, 10–13). Evidence from LMICs suggests higher rates of suicidal ideation in perinatal women compared to high-income contexts, from 14.0 to 27.5% (11, 12). Data on suicidal behavior in perinatal women in LMICs have only recently begun to emerge (4, 13, 14).

While methods exist to assess some aspects of perinatal mental health, none of the currently deployed antenatal tools are sufficiently comprehensive to explore multiple vulnerabilities. The most commonly employed tool for CPMDs is the Edinburgh Postnatal Depression Scale (EPDS) (15), a 10-item self-report measure applied throughout the perinatal period and considered reliable and valid in multiple LMICs to identify depressive and anxious symptomology (5). However, no dedicated tools for perinatal women have been designed to assess SIB. Current methods are not fit for purpose as they often inquire only about very recent changes in state, potentially missing women experiencing more chronic mental health difficulties. Further, they commonly embed questions on self-harm into tools for depression (e.g., EPDS, Patient Health Questionnaire, Beck Depression Inventory, and Hamilton Depression Rating Scale), preventing exploration of SIB in the absence of CPMDs (14). These embedded questions can inappropriately assume suicidal intent or are improperly interpreted as such by scholars with regularity, including those applying the EPDS which cannot deduce intent (3). Others conflate suicidal and non-suicidal self-harming ideation (e.g., Patient Health Questionnaire) or use vague and/or stigmatizing language (e.g., Hamilton Depression Rating Scale queries “gestures of suicide”). Still other methods explore suicidal behavior through branching logic only if suicidal ideation is reported, missing behaviors that occur under more sudden circumstances. These tools' limitations have shaped available data which is especially problematic for LMICs where the manifestation of CPMDs and links between mental disorders and SIB are less established (4). For example, SIB in the antenatal period may be predictive of postnatal depression, but reliable data are absent (14). Identifying correlates of SIB in LMIC antenatal women, particularly factors related to their life circumstances and the role of pre-conception mental health, may reveal common risk factors to CPMDs or unique variables, which could inform early intervention for CPMDs and SIB (4).

ANC in LMICs is a critical point in a woman's care-seeking to intervene for those experiencing or at risk of poor psychosocial outcomes. Coverage of ANC is nearly double that of postnatal care and roughly 86% of women attend at least one clinic during pregnancy in LMICs (16). While increasing levels of contact between pregnant women and providers are promising in LMICs, deficiencies in global guidance (17), and content and quality of visits mean ANC is an underutilized platform to generate more complete data on maternal mental health and, with the right tools, women's psychosocial vulnerabilities (18). This study capitalizes on the strengths of Sri Lanka's well-established ANC system to examine the data dearth on CPMDs and SIB among LMIC antenatal women. The Government of Sri Lanka recognizes perinatal suicides as an important public health challenge, but rigorous evidence on SIB in perinatal women is unavailable. Previous research on CPMDs in Sri Lanka has almost wholly prioritized postnatal women and excluded minority and low literacy subpopulations (3, 19, 20). As such, this research aimed to answer four research questions about the mental health of a perinatal population in Sri Lanka:

1. What is the prevalence of antenatal depressive symptomology indicative of depression?
2. What is the lifetime- and current-pregnancy prevalence of suicidal ideation and/or behavior?
3. What is the relationship between depression and SIB in this antenatal population?
4. What correlates of depression and SIB can be identified?

Through the application of an innovative screening tool including the EPDS, a modified Columbia-Suicide Severity Rating Scale, and an original Life Circumstances questionnaire, this is the first study to report findings from an antenatal population in Sri Lanka inclusive of minority women and those with low literacy, and one of the few to do so from a LMIC.

## Materials and Methods

### Case Selection and Study Setting

This cross-sectional study was conducted in Gampaha District, Western Province, Sri Lanka. Gampaha was selected as although it has historically lower suicide rates than other parts of Sri Lanka, these rates have been intractable (21). The district reports comparatively elevated numbers of perinatal suicide and a consistently higher maternal mortality ratio than the national average (55.1 vs. 33.8 per 100,000 live births, respectively) (22). Gampaha is the second most populous district with 2.4 million people, hosting a more urban, non-agricultural, and migratory population than elsewhere in the country and differing from the overwhelmingly rural populations that have centered in previous Sri Lankan research on suicide (21, 23, 24). Sri Lanka displays persistently high suicide rates with more limited declines in women (21).

Gampaha District currently delivers 185 community-based antenatal clinics (ANC) in addition to hospital-based services. ANCs selected for data collection ranged from small basic centers delivering primary maternal and child health services, to Sri Lanka's second largest public hospital, Colombo North Teaching Hospital. The combination of community- and hospital-based ANC improves representativeness (1), as hospitals often absorb higher risk pregnancies from community clinics, potentially skewing findings of hospital-only studies. This is the first Sri Lankan study to achieve this combined sample.

### Participants and Procedures

A sample size of 1,000 women was sufficiently powered to detect 15–20% antenatal depression (at 95% confidence intervals), in line with regional studies and adjusted for design effects (13, 19, 25). Sampling was a three-stage process with local health authorities, antenatal services, and individual women. Firstly, four of the District's 16 Medical Officer of Health areas were purposefully selected to ensure representativeness of the District's population density, geographical coverage, distance from referral hospitals, and patient volume. Secondly, individual community clinics were randomly selected for three Health areas. Patient load and urbanization varied across areas resulting in selection of 11 ANC services across Gampaha. Colombo North Teaching Hospital provided the hospital-based clinic in the fourth Medical Officer of Health area. Thirdly, all pregnant women aged 15–49 presenting for ANC at one of the study sites, regardless of gestation, preferred language, or literacy level were invited to participate. Women could participate only once, usually on the first occasion they attended the clinic when the research team was present.

### Measures

Three data collection tools were combined in succession to form a novel three-part instrument:

1. Edinburgh Postnatal Depression Scale

The Edinburgh Postnatal Depression Scale—producing a score between 0 and 30 (15)—has been validated to reliably detect recent depressive symptomology among both Sinhala- and Tamil-speaking populations, ante- and postnatally (26, 27). Based on local validation studies recommending cut-off scores of eight and nine for Tamil- and Sinhala-speaking populations respectively, a conservative threshold of nine and above was applied to indicate current depressive symptomology for possible antenatal depression (i.e., dysthymia through major depressive disorder). The EPDS is not a diagnostic tool, and this study does not apply the term “depression” as a formal diagnosis, but rather as a shorthand descriptor of elevated intensity and number of symptoms indicative of possible depression. Women endorsing “hardly ever,” “sometimes,” or “yes, quite often” were considered positive for past-week presence of self-harming thoughts (item 10). This single item is frequently cited as a measure of current suicidal ideation. However, its wording is broad and only asks about thoughts of self-harm in the previous 7 days. Data from previous studies have often inappropriately been interpreted as prevalence of suicidal ideation, when in fact, they could be reporting thoughts of non-suicidal self-harm. As such, a dedicated tool to isolate suicidal ideation, suicidal behavior and—crucially—non-suicidal self-harm in antenatal women was required to unpack the full range of self-directed violence antenatal women may be experiencing.

2. Columbia-Suicide Severity Rating Scale (C-SSRS)

This study is the first to adapt the C-SSRS for both a perinatal and Sri Lankan population. There are no known applications of the C-SSRS in the English-published literature from perinatal populations despite increasing concern about this group. Additionally, there are no publications on the application nor psychometric validation of the C-SSRS in Sri Lanka. However, the C-SSRS has been validated in diverse settings (28–30) and was previously translated into Sinhala and Tamil using standardized methodology for use in a researcher-administered tool (31). Given the busy ANC context in which multiple women would need to complete the tool concurrently, and limitations of technological literacy for tablet-based data collection in this setting (32), the electronic self-report version of the C-SSRS was adapted into a paper-based self-report form with expert guidance, including from Columbia University.

The C-SSRS captured data on prevalence of suicidal ideation and suicidal and non-suicidal self-harming behaviors for two time periods: women's lifetimes and current pregnancies. Community-based evidence on prevalence of self-harm is limited to one previous rural study in Sri Lanka, which did not isolate evidence for reproductive age women (23). As most women become mothers in Sri Lanka (33), application of the C-SSRS through ANC provided the best opportunity to capture reproductive age women in a community healthcare setting to build a picture of their histories of self-harm as well as assess their experiences in pregnancy. The C-SSRS was further selected because unlike most screening tools, it avoids conflating suicidal ideation with behaviors, is one of the most comprehensive instruments available, including generating evidence on non-suicidal self-harm, and may be applied with lay researchers or through self-report to measure four key constructs at two selected time points: (1) presence of suicidal ideation, (2) severity and intensity of suicidal ideation, (3) previous self-harming behavior, and (4) lethality of that behavior. Women responded to the same set of questions for both lifetime and current pregnancy and each time period was scored independently; established best practice guided scoring (34).

3. Life Circumstances questionnaire

To identify potential unique and shared correlates of depression and SIB, a third questionnaire on life circumstances was developed following a review of evidence and included sociodemographics, pregnancy and motherhood, alcohol, and marriage characteristics. A gap in evidence exists on attitudes toward and experiences of intimate partner violence (IPV) in Sri Lanka generally and among perinatal women specifically. IPV has been identified in other LMICs as a correlate of CPMDs (6), while its relationship with perinatal SIB has been explored in a handful of LMIC studies using insufficiently narrow definitions (35). This study incorporated the validated Demographic and Health Survey domestic violence module (n.d.), thrice conducted in Sri Lanka, and locally developed and validated questions assessing both justification and experience of multiple forms of IPV (i.e., physical, sexual, economic, emotional, and coercive control IPV). To our knowledge, this is one of the most comprehensive explorations of IPV's relationship with depression and SIB from any LMIC.

The C-SSRS and Life Circumstances components were translated and back-translated by two native Sinhala and Tamil speakers with expertise in psychometrics and social science research. These experts, along with two additional local (maternal) health researchers proficient in local languages, assessed back-translations for consistency in concept and interpretation across all three languages, modifying tools until consensus was achieved. The EPDS was used in its current Sri Lankan Government-endorsed format following validation studies (26, 27).

### Piloting

The instrument was piloted in February 2016 until no indications that either the content or the process of the research presented difficulty for participating women (*n* = 21). Women were asked for feedback about the screening process, given an opportunity to voice any concerns or confusion they had regarding particular items included in the instrument, remark on ease/difficulty of use, and make any additional suggestions to the research team to improve the main study and future participants' experience. No modifications were required for the EPDS, while the C-SSRS and Life Circumstances questionnaires were amended twice. The C-SSRS lethality subscale was removed after piloting due to time constraints, participant expressed challenges of recall, and as its items were deemed unessential for the aims of the research; best practice also excludes this subscale from scoring (34). Formatting and minor wording changes to retained subscales were required for both the C-SSRS and Life Circumstances components to facilitate reliable unaided self-reporting.

### Data Collection and Ethics

At each ANC, Public Health Midwives introduced the research team to attending women. Midwives acted in this introductory role for two reasons: (1) to ensure clinic operations were not disrupted by the research and (2) as midwives are familiar to antenatal women, this was viewed by the clinical and research teams as the most culturally appropriate way to support women's agency to consider their participation. Following this introduction, the research team, which consisted of the author (AP) and one of two research assistants, could directly engage with (potential) participants. AP has worked in Sri Lanka on issues of maternal and mental health and violence for more than a decade and lived in-country both during and since this study was completed. The primary research assistant was a nursing graduate with specialist training in midwifery, while the secondary research assistant was a pre-intern medical graduate; both originated from the study district. AP and one research assistant conducted data collection in parallel during each clinic. Written informed consent was provided in women's preferred language (Sinhala, Tamil, or English). Participants were given sufficient time to read the document privately and to ask any questions before deciding whether to participate. The response rate exceeded 95%; exact number of refusals could not be confirmed as midwives assisted in recruitment. Lack of time was the primary reason for refusal; no other common characteristics among those who declined were identified. Questionnaires were distributed to participants concurrently, but were self-completed privately unless support was required. Women with low literacy (*n* ~ 10) participated through oral administration of the questionnaire by the research assistant. Nearly 90% of women submitted fully completed forms which were spot-checked by the research team for completeness and indications that any participants were currently in distress (i.e., at risk of harm to self or others and/or experiencing IPV). While the EPDS and C-SSRS are accepted as safe ways to open discussions about emotions with participants (15), clear referral pathways were established. Women indicating risk were discreetly connected by the research team to their individually responsible midwife, supporting continuity of care through future home and ANC visits. Screening responses were not shared with midwives in full, rather notice of current or previous risk was disclosed in agreement with women. Materials signposting to gender-based violence or mental health services were provided to all women and clinics, and referrals made to services as appropriate through supported decision-making with women. Neither incentives nor compensation was given for participation. The London School of Economics and University of Kelaniya Research Ethics Committees granted ethical clearance, while the UK Economic and Social Research Council provided partial funding. Between February and September 2016, 1,013 antenatal women completed the questionnaire (excluding the pilot), with a minimum of 250 women from each of the four Medical Officer of Health areas.

### Data Analysis

All data were analyzed using SPSS v27.0. Thirteen cases were removed due to significant omissions. Missingness analysis concluded these cases did not show common variables or systematic errors which could have biased the remaining sample. Of the remaining 1,000 observations, 868 had complete data. As the dataset was 99.7% complete at the variable level, it was inefficient and potentially biased to proceed with analysis based on complete cases alone. Multiple imputation was not employed in this instance in alignment with scoring guidelines (34).

Internal consistency of the selected scales was assessed using Cronbach's alpha with coefficients of 0.80, 0.91, and 0.81 for the EPDS, C-SSRS, and IPV scales, respectively. These coefficients are sufficiently strong to suggest each scale exhibited adequate internal consistency and reliability in the study sample, with redundancy of items avoided. Normality of data was examined using the Shapiro–Wilke's test. Data were explored for outliers and skewness to inform appropriate test selection of parametric tests. Presence of multicollinearity was assessed. Bivariate analyses applying Fisher's exact or chi-square test of independence, *post-hoc* analyses using the Bonferroni correction and accounting for cells with few observations, were run in order to inform selection of variables for multivariate analyses.

A second set of multivariate analyses were conducted using logistic regression to examine risk factors for antenatal depressive symptomology and current-pregnancy SIB with variable selection based on bivariate analyses. Analyses sought to achieve the most parsimonious models informed by *a priori* and *a posteriori* factors. A threshold for statistical significance was set as *p* < 0.05; variables demonstrating significance in bivariate analyses were retained in multivariate models, with strength of association reflected in adjusted odds ratios (95% CI). Sensitivity analysis, Hosmer–Lemeshow's test and Nagelkerke's *R*^2^ further informed model selection, with the latter two assessing goodness of fit for the logistic regression model for depressive symptomology. Due to sparse data bias for the outcome of SIB in pregnancy, traditional methods of logistic regression risked producing a biased model. Based on the literature, Firth logistic regression was selected to address this issue (36).

## Results

### Participant's Life Circumstances

One thousand antenatal women, ranging from 16 to 42 years, participated. The majority were at least 26 years of age (66%), Sinhala Buddhist (75.9%), and achieved a minimum of some secondary school education (78.2%). Marriage was nearly universal in our sample (96.5%), and 17.7% of women married as teenagers. Child marriage (i.e., <18 years) was reported by 55 (5.5%) women. Over a quarter of women engaged in part- or full-time work outside the home (27.8%), while 44.3% were housewives. Eighty-two percent of women had partners in part- or full-time employment. One in four women had household debt, and almost half of those with debt reported that it caused worry or stress (*n* = 115/251). Aside from debt, 13.3% of all women reported their general household financial situation caused worry. Average gestation was 21 weeks (SD = 9.4), however women were sampled between 2 and 40 weeks gestation. Nearly half (45.8%) of women were attending ANC for their first pregnancy. Some women expressed ambivalent or changeable feelings about the pregnancy and did not intend (12.5%) or want (9.1%) the pregnancy. Two-thirds of women reported that their husbands drank alcohol (*n* = 617), nearly 13% of these women qualified this drinking as problematic.

A high proportion of women (43.3%) justified physical IPV for at least one of five hypothetical scenarios. Women's reported experiences of different types of IPV in their current partnership varied widely, with 1 in 4 women reporting both jealous/angry behavior from their partner if they spoke with other men (23.7%) and partners insisting on knowing their movements at all times (25.9%). One in six women reported emotional abuse (*n* = 164), while physical abuse affected 12.8% of women. Economic violence and other controlling behaviors such as limiting contact with friends and family were less commonly reported forms of IPV. Forced sex and physical harm during the current pregnancy were disclosed by 2.4 and 2.9% of women, respectively.

Table 1 presents life circumstances of women, including those reporting antenatal depression (i.e., EPDS score of nine or more) and SIB in pregnancy (i.e., ideation only, behavior only, and both ideation and behavior). Risk factors identified through bivariate analyses are indicated in bold and described by mental health outcome in the following sections. As mental health outcomes did not differ significantly between community- and hospital-based participants, results are presented for a combined sample.

**Table 1:** Description of study participants and bivariate distributions for antenatal depressive symptomology and SIB in pregnancy.

		**Descriptive statistics**	**Antenatal depression** (***n* = 296)**	**Suicidal ideation and/or behavior in pregnancy** (***n* = 74)**		
		**Full sample** (***n* = 1,000)**	**Depressive group^†^** (***n* = 296)**	**Suicidal ideation (alone)** (***n* = 41)**	**Suicidal behavior (alone)** (***n* = 10)**	**Suicidal ideation and behavior** (***n* = 23)**
	**Variable**	***N*** **(%)**	***N*** **(%)**	***N*** **(%)**	***N*** **(%)**	***N*** **(%)**
**Demographics**	Age of women (mean, S.D.)	28 (±5.4)				
	**Religion**					
	Buddhist	759 (75.9)	212 (71.6)	27 (65.9)	8 (80.0)	14 (60.9)
	Catholic	132 (13.2)	50 (16.9)	9 (22.0)	0	4 (17.4)
	Hindu	42 (4.2)	18 (6.1)	5 (12.2)	0	2 (8.7)
	Other	66 (6.6)	16 (5.4)	0	2 (20.0)	3 (13.0)
	Missing	1 (0.1)	0	0	0	0
	**Ethnicity**					
	Sinhalese	897 (89.7)	256 (86.5)	32 (78.0)	8 (80.0)	19 (82.6)
	Tamil^a^	65 (6.5)	27 (9.1)	4 (9.8)	2 (20.0)	3 (13.0)
	Minority group^b^	36 (3.6)	13 (4.4)	5 (12.2)^*^	0	1 (4.3)
	Missing	2 (0.2)	0	0	0	0
**Marriage and family**	**Marital status**					
	Single	34 (3.4)	10 (3.4)	3 (7.3)	2 (20.0)^*^	3 (13.0)^*^
	Married	965 (96.5)	285 (96.3)	38 (92.7)	8 (80.0)^*^	20 (87.0)
	Divorced	1 (0.1)	1 (0.3)	0	0	0
	Widowed	0	0	0	0	0
	**Social support**					
	No support	42 (4.2)	22 (7.4)^***^	6 (14.6)^***^	1 (10.0)	3 (13.0)
	One source of support	478 (47.8)	146 (49.3)	17 (41.5)	6 (60.0)	13 (56.5)
	Two or more sources of support	480 (48.0)	128 (43.2)	18 (43.9)	3 (30.0)	7 (30.4)
	**Living situation**					
	Alone	6 (0.6)	3 (1.0)	1 (2.4)	1 (10.0)^**^	1 (4.3)
	Nuclear family	509 (50.9)	155 (52.3)	23 (56.1)	2 (20.0)	14 (60.9)
	Extended family	482 (48.2)	137 (46.3)	17 (41.5)	7 (70.0)	8 (34.8)
	Missing	3 (0.3)	1 (0.4)	0	0	0
**Socioeconomic factors**	**Stressed by debt**					
	No	136 (13.6)	235 (79.4)	32 (78.0)	8 (80.0)	14 (60.9)^**^
	Yes	115 (11.5)	61 (20.6)^**^	9 (22.0)	2 (20.0)	9 (39.1)^**^
	**Household finances cause worry**					
	No	538 (53.8)	155 (52.4)	24 (58.5)	3 (30.0)	9 (39.1)
	Yes	458 (45.8)	141 (47.6)	17 (46.0)	7 (70.0)	14 (60.9)
	Missing	4 (0.4)	0	0	0	0
**Pregnancy and motherhood**	**Trimester**					
	1st trimester	235 (23.5)	68 (22.9)	7 (17.0)	1 (10.0)	1 (4.3)
	2nd trimester	459 (45.9)	131 (44.3)	22 (53.8)	5 (50.0)	10 (43.5)
	3rd trimester	294 (29.4)	94 (31.8)	11 (26.8)	4 (40.0)	12 (52.2)
	Missing	12 (1.2)	3 (1.0)	1 (2.4)	0	0
	**Pregnancy intendedness**					
	Intended to get pregnant	833 (83.3)	226 (76.4)^**^	24 (58.5)^**^	7 (70.0)	10 (43.5)^**^
	Intentions kept changing	37 (3.7)	17 (5.7)	2 (4.9)	0	3 (13.0)
	I did not intend to get pregnant	125 (12.5)	50 (16.9)^**^	15 (36.6)^**^	3 (30.0)	10 (43.5)^**^
	Missing	5 (0.5)	3 (1.0)	0	0	0
**Personal and family health**	**Family history of mental disorder**					
	No	968 (96.8)	286 (96.6)	36 (87.8)	9 (90.0)	22 (95.7)
	Yes	30 (3.0)	9 (3.0)	5 (16.7)^**^	1 (10.0)	1 (4.3)
	Missing	2 (0.2)	1 (0.4)	0	0	0
	**Spouse use of alcohol**					
	Never	382 (38.2)	105 (35.5)	11 (26.8)	4 (40.0)	8 (34.8)
	Sometimes	588 (58.8)	170 (57.4)	22 (53.7)	5 (50.0)	11 (47.8)
	Often	29 (2.9)	20 (6.7)^**^	7 (17.1)^**^	1 (10.0)	4 (17.4)^**^
	Missing	1 (0.1)	1 (0.4)	1 (2.4)	0	0
**IPV**	**Justifies at least one scenario of IPV**					
	No	555 (55.5)	152 (51.3)	18 (43.9)	4 (40.0)	7 (30.4)^*^
	Yes	433 (43.3)	142 (48.0)	23 (56.1)	6 (60.0)	16 (69.6)^*^
	Missing	12 (1.2)	2 (0.7)	0	0	0
	**Experienced at least one form of IPV**					
	No	456 (45.6)	88 (29.7)^**^	6 (14.6)^**^	0^*^	0^**^
	Yes	539 (53.9)	207 (69.9)^**^	35 (85.4)^**^	10 (100.0)^*^	23 (100.0)^**^
	Missing	5 (0.5)	1 (0.4)	0	0	0
	**Physical IPV in pregnancy**					
	No	958 (95.8)	268 (90.5)^**^	35 (85.4)^**^	8 (80.0)^*^	16 (69.6)^**^
	Yes	29 (2.9)	19 (6.4)^**^	6 (14.6)^**^	2 (20.0)^**^	6 (26.1)^**^
	Unsure	11 (1.1)	8 (2.7)^*^	0	0	0
	Missing	2 (0.2)	1 (0.4)	0	0	1 (4.4)
**Antenatal mental health outcomes**	**Antenatal depression**					
	No	687 (68.7)	–	9 (22.0)	3 (30.0)	2 (8.7)^**^
	Yes	296 (29.6)	–	32 (78.0)^**^	6 (60.0)	20 (87.0)^**^
	Missing	17 (1.7)	–	0	1 (10.0)	1 (4.3)
	**Suicidal ideation in pregnancy**					
	No	958 (95.8)	263 (88.8)^**^	–	–	–
	Yes	41 (4.1)	32 (10.8)^**^	–	–	–
	Missing	1 (0.1)	1 (0.4)	–	–	–
	**Suicidal behavior in pregnancy**					
	No	986 (98.6)	288 (97.3)	–	–	–
	Yes	10 (1.0)	6 (2.0)	–	–	–
	Missing	4 (0.4)	2 (0.7)	–	–	–
	**Suicidal ideation and behavior in pregnancy**					
	No	976 (97.6)	275 (92.9)	–	–	–
	Yes	23 (2.3)	20 (6.8)^**^	–	–	–
	Missing	1 (0.1)	1 (0.3)	–	–	–

a *Includes Sri Lankan and Indian Tamil.*

b *Includes Burgher, Malay, Moor, and Other.*

† *Scores of nine or more on the EPDS qualified women as depressive, i.e., indicating likely presence of antenatal depression.*

*Bolded items are significant where*

* *p ≤ 0.05,*

** *p ≤ 0.01,*

*** *p ≤ 0.001.*

*Antenatal depression and SIB categories are reported separately in this table. Women qualifying as depressive could also be experiencing some dimension of SIB and vice versa. Co-morbidity is captured through antenatal mental health outcomes. Supplementary results of bivariate analyses for mutually exclusive categories (depression, current SIB, and co-morbid depression and SIB) are available upon request*.

### Prevalence and Correlates of Antenatal Depression

Nearly one in three women (29.6%) reported depressive symptomology indicative of antenatal depression (*n* = 296). Mean EPDS score was 6.6 (SD = 5.0; variance 0–26), with a significant difference in the mean total score between women qualifying as depressed (mean total score 12.7, SD = 3.7) vs. non-depressed (mean total score 4.0, SD = 2.5; *p* < 0.05).

Drawing on results of bivariate analyses (Table 1), logistic regression isolated risk factors for antenatal depression in this setting. Table 2 presents adjusted odds ratios (aOR) (95% confidence intervals). Women with secondary education were 34% less likely to have antenatal depression compared to women with only primary education (*p* = 0.05). Women were significantly more likely to have depression if their spouse was unemployed (aOR 4.57, *p* < 0.05), and being stressed or worried by debt was on the cusp of being significantly associated (aOR 1.61, *p* = 0.06). Family history of mental disorder and justifying IPV rendered women less likely to have depression, while all forms of experienced IPV, except forced sex (i.e., marital rape), were significantly correlated with depression (*p* < 0.05). Women who felt their husbands engaged in problematic drinking were twice as likely to qualify as having antenatal depression (aOR = 2.21, *p* < 0.001). Spousal unemployment along with lifetime history of suicidal ideation, suicidal behavior, or both were the strongest correlates of depressive outcomes. Those with a lifetime history of both suicidal ideation and behavior were nearly nine times more likely to exhibit antenatal depression as women without (*p* < 0.001).

**Table 2:** Risk factors of antenatal depressive symptomology (adjusted odds ratios) (*n* = 296).

**Variables**	**aOR**	**95% CI**		***p*-value**
		**Lower**	**Upper**	
**Age**				
15–19^*^				
20–25	0.96	0.47	1.95	0.91
26–34	1.15	0.58	2.27	0.69
35–49	1.04	0.48	2.29	0.91
**Ethnicity**				
Sinhalese^*^				
Tamil (SL or Indian)	1.48	0.77	2.87	0.24
Minority group (Burgher, Malay, Moor, Other)	0.49	0.18	1.32	0.16
**Education**				
Primary^*^				
Secondary	**0.66**	**0.44**	**1.00**	0.05^*^
Higher education	0.61	0.34	1.10	0.10
**Worried by debt**	1.62	0.99	2.67	0.06
**Spouse's employment**				
Full- or part-time employed^*^				
Unemployed	**4.57**	**1.20**	**17.50**	0.03^*^
Other	0.73	0.46	1.16	0.18
**Spouse's drinking problematic**				
No^*^				
Yes	**2.21**	**1.47**	**3.34**	0.00^*^
Unsure	0.80	0.34	1.88	0.62
**History of mental health**				
Family history of mental disorder	**0.31**	**0.11**	**0.87**	0.03^*^
Lifetime history of only SI	**3.49**	**2.27**	**5.36**	0.00^*^
Lifetime history of only SB	**9.09**	**1.95**	**42.27**	0.01^*^
Lifetime history of both SIB	**8.73**	**4.79**	**15.92**	0.00^*^
**Justifies IPV in event of perceived child neglect**				
No^*^				
Yes	**0.64**	**0.44**	**0.92**	0.02^*^
Unsure	1.08	0.62	1.87	0.79
**Spouse is jealous or angry**				
No^*^				
Yes	**1.69**	**1.14**	**2.51**	0.01^*^
Unsure	1.16	0.66	2.04	0.60
**Spouse limits family contact**				
No^*^				
Yes	**3.09**	**1.34**	**7.16**	0.01^*^
Unsure	2.82	0.67	11.86	0.16
**Spouse doesn't trust her with money**				
No^*^				
Yes	**2.12**	**1.24**	**3.62**	0.01^*^
Unsure	2.04	0.77	5.42	0.15
**Experienced physical IPV**				
No^*^				
Yes	**1.75**	**1.04**	**2.93**	0.03^*^
Unsure	2.63	0.28	24.71	0.40
**Experienced forced sex by spouse**				
No^*^				
Yes	0.36	0.11	1.12	0.08
Unsure	1.58	0.16	15.82	0.70

* *Denotes reference category. Significance p ≤ 0.05 for bolded and starred items. Hosmer-Lemeshow Chi-square is 8.28 and p = 0.41 (>0.05). Nagelkerke R^2^ is 0.29. The model correctly classifies 77.5% of cases*.

### Self-Harming Ideation and Behavior in Women's Lifetimes and Current Pregnancies

A quarter of women had a lifetime history of SIB (i.e., ideation only, behaviors only, or the combination of both experiences) (25.7%), while current pregnancy prevalence of SIB was 7.4%. Lifetime- and current-pregnancy suicidal ideation which did not escalate to subsequent behavior was reported respectively by 14.9 and 4.1% of women. Women reporting only suicidal behaviors (i.e., without the co-occurrence of suicidal thoughts) were disaggregated to explore potential sudden acts: 12 women (1.2%) reported this in their lifetimes and 10 (1.0%) during their current pregnancy. Nearly 11% (*n* = 108) of women reported at least one form of suicidal behavior in their lifetime including a suicide attempt (*n* = 86), interrupted (*n* = 43), or aborted attempt (*n* = 51) or preparatory/rehearsal behaviors (*n* = 39). Non-suicidal self-harm was reported in 6.4% of women ever in their lifetime. Current pregnancy prevalence of at least one form of suicidal behavior stood at 3.3%, three-quarters of which were suicide attempts (*n* = 24), while 1.9% of pregnant women (*n* = 19) disclosed non-suicidal self-harm during their current pregnancy.

Prevalence of any form of self-directed violence—whether suicidal or non-suicidal in nature—was therefore higher than looking at intent categories in isolation, with 12.9 and 4.0% of women endorsing self-harming behavior regardless of intended outcome in their lifetimes and pregnancies, respectively. Ultimately 11.7% of women (*n* = 117) required urgent referral based on endorsement of intent and plans to act on suicidal ideation (*n* = 64), endorsement of EPDS item 10 (*n* = 95), and/or reporting of a suicidal or non-suicidal self-harming episode in their current pregnancy.

Bivariate analyses explored women's life circumstances as potential correlates of SIB in pregnancy, with younger age (*p* < 0.001), being an ethnic minority (*p* < 0.05), being unmarried (*p* < 0.001) and whether the woman was married as a child (*p* < 0.001) all significantly related. Husband's employment status was not associated with SIB in pregnancy. The household being in debt (*p* = 0.05), perceived stress from both debt and general household finances (*p* < 0.01), perceived total lack of social support (*p* < 0.001), living alone (*p* < 0.05), unplanned and/or unwanted pregnancy (*p* < 0.001), husbands drinking “often” (*p* < 0.001), and drinking being viewed as problematic (*p* < 0.001) were associated with SIB in pregnancy. Family history of mental disorder and a lifetime history of SIB were significantly correlated with SIB in the current pregnancy, as was EPDS item 10 and total EPDS score (all *p* < 0.001). As with depression, justifying wife beating in the event of a wife arguing with her husband (*p* < 0.05) and all forms of experienced IPV were strongly related to women's current SIB. Multivariate analyses using Firth logistic regression isolated risk factors of SIB in pregnancy (Table 3).

**Table 3:** Risk factors of suicidal ideation and/or behavior in pregnancy (adjusted odds ratios) (*n* = 74).

**Variables**	**aOR**	**95% CI**		***p*-value**
		**Lower**	**Upper**	
**Education**				
Primary^*^				
Secondary	0.55	0.24	1.27	0.16
Higher education	0.27	0.06	1.22	0.09
**History of mental health**				
Family history of mental disorder	3.56	2.25	15.63	0.09
Lifetime history of both SIB	**10.69**	**4.47**	**25.56**	0.00^*^
**Antenatal mental health outcomes**				
Antenatal depression present*^†^*	1.90	0.84	4.34	0.13
Self-harming thoughts in past week	**9.61**	**4.26**	**21.70**	0.00^*^
Current pregnancy non-suicidal self-harm	**17.27**	**3.08**	**96.84**	0.00^*^
Experienced any form of IPV	**4.36**	**1.33**	**14.35**	0.02^*^

* *Denotes reference category. Significance p ≤ 0.05 for bolded and starred items. Hosmer-Lemeshow Chi-square is 11.18 and p = 0.19 (>0.05). Nagelkerke R^2^ is 0.60. The model correctly classifies 95.1% of cases.*

† *Scores of nine or more on the EPDS indicate likely presence of antenatal depression*.

Women's overall EPDS score was not significantly associated with current pregnancy SIB in our sample once other factors were taken into account (aOR = 1.90, *p* = 0.13). However, responses to item 10 on the EPDS indicating presence of self-harming thoughts in the previous week (aOR = 9.61) and disclosure of non-suicidal self-harming behavior in the current pregnancy (aOR = 17.27) were strongly correlated with SIB in pregnant women (*p* < 0.001). Women who experienced at least one form of IPV in their current partnership were four times as likely to report current SIB (*p* < 0.05) and lifetime history of suicidal ideation, suicidal behavior or both increased risk of SIB in pregnancy by 11 times (*p* < 0.001).

## Discussion

Viewing ANC attendance as a window of opportunity to explore multiple psychosocial vulnerabilities in women, our study revealed depression and SIB in antenatal women in urbanizing Sri Lanka to be common. We found that nearly one in three women demonstrated high depressive symptomology, which is above the 16–25% pooled prevalence of antenatal depression observed across other LMICs (6) and substantially higher than previous studies in Sri Lanka (19, 20) with exception of one recent report of 26.5% prevalence in a very limited antenatal population (3). Our finding may reflect our more representative sample which included Tamil-speaking mothers, those with low literacy and from all trimesters previously excluded from Sri Lankan research and both hospital- and community-based women. Community ANC is nearly universal in Sri Lanka, and women attend an average of 5.6 appointments per pregnancy in Gampaha (22). Furthermore, the rapidly urbanizing context of this study where migration levels are high may play an underlying role in the high rates of depression observed compared to more traditional and rural parts of Sri Lanka where extensive social support networks may be more accessible due to women's proximity to their natal homes (19).

Correlates of depression identified in this study have been found among perinatal populations in other LMICs: lower education (6, 20), lack of social support (10), and having an unplanned and/or unwanted pregnancy (1). Growing evidence explores the role of poverty and deprivation in perinatal mental health and SIB (37). Researchers have measured this evidence objectively and subjectively by socioeconomic status, income, occupation, food insecurity, assets, and debt (4, 23). In this study, women's depression was associated with husbands' unemployment and the subjective experience of being worried by debt and broader household financial difficulties in line with other LMIC research (25). In Sri Lanka, the role of deprivation in perinatal women's mental health has not previously been explored, but unemployment is associated with SIB in men (23). As women are heavily financially dependent on men in South(east) Asian contexts, spousal employment may indirectly affect women's mental health in pregnancy. Husbands' alcohol use and women's perceptions of problem drinking in a spouse were associated with depressive outcomes which has also been found in other LMIC and non-pregnant women in Sri Lanka (38). Alcohol consumption among women is exceedingly low in Sri Lanka, while rates of alcoholism in men are high for South Asia and rising (24). There is evidence that alcohol abuse in men works indirectly to impact women's mental health by exacerbating other life stressors (24). We did not find age, marital characteristics, ethnicity, living arrangement, son preference, or parity to be related to antenatal depressive symptoms although these have been observed elsewhere (25). Sri Lanka does not have a strong history of son preference nor sex-selective abortion as seen in other Asian contexts. As abortion remains heavily restricted in Sri Lanka, disclosure rates in this sample were too low to meaningfully explore its role in women's mental health outcomes.

The innovative use of the C-SSRS during the antenatal period contributes to the literature in four ways. Firstly, as a separate instrument, the C-SSRS independently assessed prevalence of suicidal ideation, suicidal behavior, both experiences and non-suicidal self-harming behavior in women, without conflating them with depression or reducing them to just one item—unlike commonly used screening tools. Total EPDS score was not significantly associated with antenatal SIB once other factors were considered. Although a majority of women experiencing SIB in pregnancy reported co-morbid depression, one in five women disclosing current-pregnancy SIB did so in the absence of antenatal depressive symptomology. The possibility of SIB *exclusive* of depression echoes recent research from South Africa, but contrasts with evidence from high-income countries asserting SIB only occurs in the context of major mental disorder (4). This study therefore challenges the dominant psychiatrized Western discourse and instead suggests depression is not a pre-requisite for SIB in pregnancy in this setting. Separate dedicated tools are thus preferable to assess both phenomena independently to ensure women experiencing SIB without co-morbid CPMDs are not missed.

Secondly, as the C-SSRS captures risk from two time points, it maximizes the opportunity of women's attendance at ANC to generate data on their mental health from outside the pregnant experience by screening for lifetime SIB, addressing a gap in evidence on community-based samples of reproductive age women in Sri Lanka. It cannot be assumed that 25.7% lifetime prevalence of SIB is representative of all women of reproductive age in this context, as there may be differences in SIB between women who go on to become mothers and those who do not. However, like many LMIC, most women in Sri Lanka bear children as childlessness remains heavily stigmatized while achieving motherhood fulfills gendered marriage expectations, with pressure to conceive soon after wedding (24, 33).

Thirdly, by exploring two time points, significantly lower prevalence of SIB in pregnancy (7.4%) is observed compared to women's whole lives (25.7%), suggesting pregnancy may be “protective” against SIB in Sri Lanka (14). This protective effect has not been observed in all settings, in particular high-income countries where suicidal ideation presents similarly between pregnant and non-pregnant populations (39). Previous LMIC research found higher prevalence of perinatal suicidal ideation (14.0–27.5%) compared to our sample (4, 11, 12), however, this study closely mirrors recent research from a similar cultural context in urban south India (13). The variability in prevalence seen across contexts is likely due to selected instruments and definitions of SIB employed in studies, among other contextual factors. Despite this possible pregnancy protective effect, nearly 12% of the sample required urgent referral based in part on current risk from self-harming ideations and/or behaviors. Finally, lifetime SIB was strongly associated with both depressive and current pregnancy SIB outcomes as seen in other LMIC settings (3, 13). This supports global evidence that a history of suicidal ideation and particularly behavior is a critical factor identifying those most at risk of later suicide (9), and indicates women's experience during pregnancy and beyond may be influenced by pre-pregnancy difficulties. Inclusion of items on women's history of SIB and past and present non-suicidal self-harm would likely improve upon currently employed screening tools for perinatal mental health.

This study reports the first estimates for Sri Lanka of the prevalence and role of IPV in antenatal depression and SIB, which emerged as a critical vulnerability in antenatal women. Multiple forms of IPV were associated with antenatal depressive outcomes as seen in a range of settings (1, 6, 10, 40). Women exposed to IPV were four times more likely to report SIB in pregnancy, echoing observations elsewhere (4, 10, 13). Prevalence of physical abuse during the current pregnancy was lower (3%) than expected based on recent lifetime physical IPV rates from Sri Lanka of 19% (41). However, it mirrors low prevalence observed in both a single study exploring physical violence in pregnancy in rural south-eastern Sri Lanka (4.7%) and a recent national study on violence against women in which 6.5% of ever-pregnant women in the sample reported physical IPV during the antenatal period (41, 42). Yet crucially, all 33 women reporting suicidal behavior during pregnancy experienced IPV, as did 85% of women with antenatal suicidal ideation. It may be that pregnancy affords women some respite from violence in their relationships in this context, which may or may not return post-birth. Experiences of forced sex were associated with depression in bivariate analyses, but not once other factors were considered, in part because women were more likely to say they were “unsure” whether they had experienced forced sex. This may reflect current cultural and legal frameworks in Sri Lanka, as marital rape is unpunishable under law. Sex within marriage is still commonly viewed as the “right” of the husband, although changes to the Penal Code are repeatedly promised then recanted (33, 43). Justifying physical IPV in particular circumstances appeared to reduce the likelihood of observing antenatal depression. However, this could reflect deeply entrenched marriage norms to excuse inappropriate behavior of husbands and silently tolerate conflict (24), minimizing recognition or disclosure of violence's impact on mental health. As IPV appeared strongly associated with maternal mental health in this setting and holds consequences for adverse outcomes for both mother and child (2), this study advocates for routine assessment of IPV in the antenatal period.

This research has several limitations. Despite a very high response rate, it is possible that non-responders were qualitatively different from those who accepted. Pressures of social and cultural acceptability and stigma may have impacted disclosure levels, but both mental health and IPV prevalence rates in this study are likely to be under-, not over-estimates, reinforcing the importance of our findings (40). As this study was conducted in one district, replication in diverse localities across Sri Lanka would be beneficial to assess the generalizability of findings. No studies have been done in Sri Lanka to validate the full or abridged version of the C-SSRS nor has it been validated among perinatal women in any global context, and future assessment of the Scales' psychometric properties is welcome. However, this study's self-report version underwent thorough cultural and linguistic adaption and piloting processes with guidance from Columbia University and local psychometrics experts. The proportion of women experiencing any form of SIB during pregnancy did not support multivariate analyses disaggregated by dimension. Finally, this study was cross-sectional, so neither causality nor postnatal mental health outcomes could be assessed. Most correlates considered, however, would have temporally preceded women's pregnancies suggesting possible directionality. Future research would benefit from longitudinal exploration of women's pre-conception vulnerabilities through postnatal outcomes, specifically to assess relapses in risk for IPV and SIB.

## Conclusion

Our analyses point strongly to the need for prioritization and more comprehensive assessment of women's psychosocial vulnerabilities in pregnancy in LMICs and contribute to emerging research on promising methods to explore psychosocial health and distress among LMIC women more broadly (18). Given ANC's effectiveness in delivering integrated services and its high rates of use in LMICs and Sri Lanka in particular, it is a potentially powerful platform through which to provide psychosocial screening inclusive of validated measures for CPMDs, SIB, and IPV. The act of screening itself has demonstrated multiple positive impacts on maternal mental health in antenatal women in LMICs (44), but should be done with the intention and capacity for onward referral to relevant support. Recognizing limitations facing resource-strained health systems across LMICs and user- and provider-level barriers to ANC and appropriate screening programs (2, 17), the Sri Lankan experience highlights opportunities to systematically address maternal mental health.

As of 2012, the EPDS was incorporated in the national pregnancy care program for the postnatal period, with some discussion to expand its use in antenatal women. Though implementation of postnatal screening is still piecemeal, awareness of the essentiality of supporting maternal mental health is growing (43). Drawing on trauma-informed and women-centered models of care (45), screening for IPV and maternal mental health could be explored as an additional, but core component of routine ANC visits. While this integration is not without its challenges (17), it is first and foremost about providing women with a safe and empathetic environment in which to disclose past or present distress. Building on the strengths of the antenatal platform, service providers may explore sensitive and pragmatic mechanisms to support improved maternal mental health and psychosocial outcomes for women and their families.

## Data Availability Statement

The raw data supporting the conclusions of this article will be made available by the authors, without undue reservation.

## Ethics Statement

This research was approved by the London School of Economics and Political Science Research Ethics Committee, the University of Kelaniya's Faculty of Medicine Ethics Review Committee (Ref. No. P/135/08/2015), the Regional Director of Health Services — Gampaha District, and the Director and Assistant Director of Colombo North Teaching Hospital. All study participants provided written informed consent before data collection commenced.

## Author Contributions

AP conceived the project, designed, coordinated and co-conducted data collection, conducted all analyses and writing of this report, and acquired funding for the study.

## Conflict of Interest

The author declares that the research was conducted in the absence of any commercial or financial relationships that could be construed as a potential conflict of interest.
